# Are treatment supporters relevant in long-term Antiretroviral Therapy (ART) adherence? Experiences from a long-term ART cohort in Uganda

**DOI:** 10.1080/17441692.2018.1514418

**Published:** 2018-08-26

**Authors:** Sarah Nakamanya, Billy N. Mayanja, Richard Muhumuza, Dominic Bukenya, Janet Seeley

**Affiliations:** a MRC/UVRI and LSHTM Uganda Research Unit, Entebbe, Uganda; b London School of Hygiene and Tropical Medicine, London, UK

**Keywords:** Treatment supporters, viral load, long-term ART, ART adherence, Uganda

## Abstract

Background: This study aimed to understand the relevance of treatment supporters in adherence among people living with HIV taking Anti-retroviral therapy (ART) for more than five years in Uganda. Methods: In-depth interviews were conducted with 50 participants (28 women and 22 men) of the Complications of Long-Term ART (CoLTART) cohort with experience of at least five years on ART in Uganda. Participants were stratified by line of ART regimen and viral loads of less or above 1000 copies/ml. Data were analyzed thematically. Results: Many participants felt that a treatment supporter was most useful at the beginning of therapy before individuals get used to the drugs or when they are still weak. However, this did not reflect treatment outcomes, as many individuals without treatment supporters had failed on first line ART regimens and were switched to second line ART. Those who were still on first line had viral loads of ≥1000 copies/ml. There was a preference for female treatment supporters, many of who were persistent in their supportive role. Conclusion: Treatment supporters remain important in adherence to long-term ART. HIV-care providers need to encourage the involvement of a treatment supporter for individuals taking ART long-term.

## Background

The introduction of antiretroviral therapy (ART) into HIV care and treatment, has restored health to many people living with HIV (PLHIV)(Gusdal et al., 2011; Igumbor, Scheepers, Ebrahim, Jason, & Grimwood, 2011; Joint United Nations Programme on HIV/AIDS, 2016). In 2013, it was reported that the scaling up of ART had averted an estimated 4.2 million deaths in low- and middle-income countries within a period of a decade (World Health Organisation, 2013). However, all these ART-related benefits are dependent on good adherence (Loutfy et al., 2013; Mayanja et al., 2013; Robbins, Spector, Mellins, & Remien, 2014).

Retaining people on ART in care and ensuring good treatment adherence are critical for long-term viral load suppression (Li et al., 2014; Viswanathan et al., 2015). When HIV viral loads are suppressed, individuals are likely to remain clinically stable and HIV transmission significantly reduced (Loutfy et al., 2013; O'Connor et al., 2017). However, to achieve optimal HIV viral suppression, adherence levels of 90% to 95% are required (particularly for older ART regimens, which continue to be used in parts of sub-Saharan Africa).

To encourage patients’ adherence to ART in sub-Saharan Africa, several approaches have been used: intensified adherence counselling, reminder alarms/calendars, daily or weekly mobile phone text messages, electronic pill devices, adherence clubs and the use of treatment supporters (also called treatment buddies or medicine companions)(Church et al., 2015; Hirnschall, Harries, Easterbrook, Doherty, & Ball, 2013; Knodel, Hak, Khuon, So, & McAndrew, 2011; Kunutsor et al., 2011; Mills et al., 2014; World Health Organisation, 2004). A randomised controlled trial in South Africa, which followed participants for two years, reported better adherence and ART outcomes in the arm that had treatment supporters than the arm without them (Nachega, Mills, & Schechter, 2010). Similarly, a one year follow up randomised controlled trial in Rakai district in Uganda, among pre-ART patients, also reported less attrition from the care cascade, slower HIV-related disease progression and better quality of daily life in the arm with treatment supporters compared to the one without supporters (Nakigozi et al., 2015). A similar finding was observed in central Mozambique (Stubbs, Micek, Pfeiffer, Montoya, & Gloyd, 2009). A global meta-analysis reported that treatment supporters are particularly effective in promoting ART adherence (Kanters et al., 2016). Although, evidence from a cohort study in western Kenya suggests that treatment supporters help to improve clinic attendance for women but not for men (Kibaara et al., 2016).

A number of studies have shown that treatment supporters’ roles go beyond encouraging adherence to drugs, to include supporting a number of different daily needs of the person they are supporting (Bezabhe et al., 2014; Duwell et al., 2013; Nachega et al., 2006; O'Laughlin, Wyatt, Kaaya, Bangsberg, & Ware, 2012; Tuhadeleni, Gary, Ashipala, & Nuuyoma, 2016; Ware et al., 2009). While utilising existing social supportive relationships may lead to sustained positive changes in adherence behaviour (Knowlton et al., 2011), designated treatment supporters have been shown to have an important supportive role in adherence in the first three months of ART but become less essential after six months and beyond (Foster et al., 2010; Kelly, Hartman, Graham, Kallen, & Giordano, 2014; Kim, McDonald, Kim, Foster, & Fidler, 2015; Rozanova, Brown, Bhushan, Marcus, & Altice, 2015). There are fewer studies on the role treatment supporters may play over an extended period of years for a person taking ART (Etard et al., 2007; Inzaule, Hamers, Kityo, de Wit, & Roura, 2016).

ART has been available in Uganda, the setting of the study reported in this paper, for over a decade. The number of HIV-positive people on ART in Uganda has steadily increased to about 898,197 (60% coverage) by the end of June 2016 (Uganda AIDS Commission, 2016). The current guidelines for prevention and treatment of HIV in Uganda recommend the use of treatment buddies in adherence support strategies (Ministry of Health Uganda, 2016). In this paper, drawing on qualitative data, we investigate the role of treatment supporters in sustaining adherence for people living with HIV on long-term ART.

## Methods

### Study population and setting

This was a cross-sectional qualitative study conducted in Wakiso and Kalungu districts in central Uganda. This study was nested in the Complications of Long-Term Antiretroviral Therapy (CoLTART) cohort study. CoLTART was a three-year cohort established in 2013 to study the long-term clinical and virological consequences of ART and HIV among African patients (Mayanja et al., 2017). The CoLTART study participants in Kalungu district were the former participants in the Rural Clinical Cohort (RCC) in Kyamulibwa which was initially established to study the natural history of HIV infection. ART was introduced among these participants in 2004 (Mayanja et al., 2013). The CoLTART participants in Wakiso district were drawn from the former Entebbe site of the Development of Antiretroviral Therapy in Africa (DART), a multisite, randomised clinical trial monitoring strategy for the management of ART in adults with HIV infection in Africa (DART Trial Team, 2010). ART was initiated in 2003 among the DART trial participants. ART eligible participants in both these two cohorts (RCC and DART) were offered pre-ART health education, including information on the importance of adherence to ART. They were also provided with continuous ART health education at every ART drug refill appointment. At the time of initiation on ART in both the RCC and DART participants were required to present a treatment supporter as one of the prerequisites for ART initiation. The CoLTART study, which brought together members of both these cohorts provided us with a platform to investigate adherence trajectories and PLHIV’s perceptions of treatment supporters’ relevance in adherence to long-term ART.

Between July 2013 and August 2014, the CoLTART study enrolled 953 individuals who had been on ART for 6 or more months, of whom 119 (12.5%) had HIV viral loads (VL) of ≥ 1000 copies/ml and 110 were sequenced for drug resistance testing, 73 (66.4%) were female, 95 (86.4%) were aged 35 years and above, 89 (80.9%) had been on ART for nine or more years, with 74 (67.3%) on first and 36 (32.7%) on second-line ART regimens at enrolment (Namakoola et al., 2016). The 2016 WHO and Uganda consolidated guidelines on the use of antiretroviral drugs for treating and preventing HIV infection both define a viral load threshold of <1000 copies/mL as successful viral suppression (Ministry of Health Uganda, 2016; World Health Organisation, 2016).

### Sampling, data collection, management and analysis

Fifty participants selected from the 953 who had been on ART for six months or more participated in this qualitative study. These were selected using a random stratified sampling technique. The qualitative study participants included those on either a first or second line ART regimen with a viral load of <1,000 or ≥1000 copies/ml. In addition, we chose both men and women and a wide age range (23–66 years), to generate a variety of experiences. The sample comprised participants who had been on ART for at least five years and beyond and these were evenly distributed between the Wakiso and Kalungu district CoLTART cohort study sites. It should be noted, that whether the participant had a treatment supporter was not a selection criterion.

### Data collection

From September 2015 to April 2016, two experienced interviewers collected data using in-depths interviews. Topics covered included experiences of HIV treatment, pathways from testing through ART treatment initiation, retention or attrition from care, life on ART, barriers and facilitators to ART adherence and treatment support. Participants who reported having a treatment supporter were asked about the nature of the relationship they had with the person, if the supporter had been considered more useful at sometimes rather than at others and when the supporter last reminded or checked on them regarding ART adherence. The interviews were conducted in the local language, Luganda, and audio-recorded, transcribed and translated verbatim into English. Data analysis was conducted by the two interviewers in collaboration with three other team members. A thematic coding framework was developed basing on the study data and themes derived from the initial reading of interviews. This framework was used for coding the study data, with constant comparison across the team to ensure consistency in coding. Thematic matrices were developed from the data, for each main theme, and summaries of the findings were developed. These summaries, and analytical memos of reflections on the findings during coding, provided rich information on treatment supporters, the focus for this paper.

### Ethical considerations

The Uganda Virus Research Institute Research and Ethics Committee (UVRI REC) and the Uganda National Council for Science and Technology (UNCST) approved this study. Prior to the interviews, written signed (or witnessed thumb-printed in case of non-literate participants) informed consent was gained. All agreed to anonymised quotes from their interviews being used in publications. Participants were compensated for their costs associated with participation.

## Results

Most of the participants had attained primary level education or below, with farming or fishing being the main occupations (Table 1).

**Table 1.** Participants demographic characteristics.

Variable	Total (*n* = 50) N (%)	Females (*n* = 28) N (%)	Males (*n* = 22) N (%)
Mean age, years (range)		42.0 (23–67)	42.5 (25–66)
Education^a^			
Primary and below	35 (70)	17 (61)	18 (82)
Secondary	9 (18)	6 (21)	3 (14)
Tertiary	6 (12)	5 (18)	1 (5)
Marital status			
Married/cohabiting	28 (56)	11 (39)	17 (77)
Single/separated/widowed	22 (44)	17 (61)	5 (23)
Religion^a^			
Christian – Catholics	24 (48)	11 (39)	13 (59)
Christian -- Anglicans	9 (18)	6 (21)	3 (14)
Christian -- Pentecostals	4 (8)	1 (4)	3 (14)
Moslems	7 (14)	6 (21)	1 (5)
Other	6 (12)	4 (14)	2 (9)
Ethnicity			
Baganda	33 (66)	20 (71)	13 (59)
Others	17 (34)	8 (29)	9 (41)
Employment^a^			
Formal employment	5 (10)	3 (11)	2 (9)
Business	15 (30)	10 (36)	5 (23)
Farming/fishing/other	30 (60)	15 (54)	15 (68)

^a^Total percentages do not add up to 100% due to effect of rounding to nearest whole number.

Participants had to present a treatment supporter on ART initiation but much as it was a prerequisite, individuals were not denied ART if they failed to present a supporter. Seventy percent (35) of the participants (18 females and 17 males) had presented with a treatment supporter at ART initiation. Of the 15 participants who never presented a supporter, five reported just giving names and telephone contacts of the people they expected to help them when they had initiated ART. While seven others did not have anyone to present, or feared to tell someone why they needed a supporter due to lack of trust in the people who might be available, having no body to ask, or living alone:

‘With the one I wanted to present, I feared that she could spread it [information on the participant’s HIV-status]’ (female aged 23 years, second line ART, VL ≥ 1000 copies/ml, been on ART for 5 years).

The remaining participants claimed their children reminded them, so they had no need for a formal ‘treatment supporter’.

### Treatment supporter’s role during the early years on ART

Most participants noted that a treatment supporter was more important when they were just starting ART because, they said, they were new to the practice of drug taking and had not got used to daily pills. Others noted that at the time of their ART initiation, they were very weak and could spend most of the time sleeping, so they needed to be woken up to take their pills. Some said they feared taking the drugs because they had experienced bad side effects like hallucinations and did not want to take the medicine again, so needed support. All these experiences, mostly during the early days on ART, meant that having a treatment supporter was of great value:

… when I am at home, she (treatment supporter) reminds me though I have now got used […]. At the beginning, she was very useful because I used to forget and she would bring me the drugs to the garden. She went on drugs before me so we would both remind each other (male aged 44 years, second line ART, VL <1000 copies/ml, on ART for 9 years).

A few participants, however, did not agree that treatment supporters were more useful in the initial days of ART.

Yes, you can get used like you have to pray before you sleep but because you have another person who knows that you are supposed to pray, that person can remind you when you have forgotten. If someone comes and tells you that please it is eight o’clock and you need to take your medicine, that person has helped you a lot. So, you can’t tell me that when you are used you don’t need that person a hundred percent (male aged 44 years, second line ART, VL ≥ 1000 copies/ml, on ART for 8 years).

Others, like Phillip (pseudonym) a 37-year-old man on second line ART at the time of the interview (with a viral load above 1000 copies/ml), had been on ART since 2004 and had adhered well in the early years. He told us how he had continued to depend on his treatment supporter, particularly when he had had a period without ART. Phillip who had never married, lived near his mother who was his treatment supporter. There was a time when he was imprisoned because of a land-dispute. After he was released he went to another district until the situation calmed down. Since that place was far from his home, he finished his supply of drugs while he was away. He was off ART for many months. When he returned and resumed ART, the drugs failed to work, so he had to switch to second line ART. His mother reprimanded him for his poor adherence but then relented and resumed her supportive role.

Every morning, she would remind me to take my pills. Now that I built just next to her house I take breakfast at her place (house). I also decided to keep my drugs at her place so that I swallow them in front of her so as to do away with that quarrel [over whether I had taken them].

### Treatment supporter’s role in long-term ART

Four other participants explained that a treatment supporter’s role comprised of comforting, especially in cases when one was stressed and could easily forget or discontinue the drugs. These participants said that after being on ART for a long time, problems like physical pain or sickness disappeared completely but then other non-medical problems occurred, and stress continued. Such problems included children’s school fees, family issues and other socioeconomic challenges, thus causing worry, which consequently affected adherence:

A treatment supporter should have been there all the time because life changes. Today you are happy, tomorrow you are not; and if someone is not there for you, or doesn’t really understand you, he/she will never help you. I remember when I lost my sister, it was so untimely. When they called me, I just rushed, and then my mum asked me if I had brought my drugs. Sincerely I had forgotten them, then I had to send somebody back. You know when I was coming; they called me on the way that she had died, I thought I was going back. So surprisingly, after all preparations for the funeral, my mum whispered to me to ask if I had carried all my drugs. (female aged 43 years, second line ART, VL ≥1000 copies/ml, on ART for 10 years)

Some participants reported that treatment supporters remained useful to long-term ART adherence particularly when they had got tired of taking the pills; it was their treatment supporter who had urged them to continue.

‘My treatment supporter has helped me a great deal in my drug taking because she has been my comforter especially in moments when I feel distressed and feel like giving up on drugs and die. My treatment supporter covers this gap because she always encourages me and gives me the strength to carry on (female aged 37 years, first line ART, VL <1000 copies/ml, on ART for 5 years)

Sometimes individuals found it difficult to manage the pill burden particularly if they were coping with other illnesses. There were a few older participants (over 60 years) who reported forgetting whether they had taken their drugs or not, and needed help in remembering to take their pills. Younger participants also talked of the value of having someone to remind them, long after they had initiated ART. For example, there was a 51-year-old man who said that he had become busy with daily life and he would sometimes wonder whether he had taken his drugs or not. He would in such cases consult his wife, who was his treatment supporter. Unfortunately, if he had been out working in a different place from her, she would not know if he had taken his pills, so she could not always help him.

### Choice of a treatment supporter

In choosing a treatment supporter, participants usually considered things like the trust they had in the person while others chose those people who had encouraged them to take an HIV test. In some cases, they chose people they lived with under the same roof, for example, a partner or other close family member. For those who lived alone, they chose a relative or friend who lived nearby. In many cases, these friends were also on ART. However, of the 10 participants who reported continued support from their treatment supporter, only six still lived with or near the treatment supporter that they had presented at ART initiation. The other four participants would receive telephone calls to check on whether they were taking the drugs.

Men who lived with a wife often presented her as their treatment supporter. Men who had got new partners after starting ART reported that their new wife had taken on an informal supportive role. Women who were treatment supporters not only reminded their partners to take their medication but also provided care by ensuring food was available when the drugs needed to be taken and making efforts to ensure their husband stayed well. However, there was one married man who had presented his wife as his treatment supporter who reported that she had been of no help and had never reminded him to take his ART.

Women seldom presented their resident husbands as their treatment supporters. Women were more likely to present a child/mother/sister or a friend as treatment supporter regardless of whether they lived with their partner or not. Most women chose a female supporter (14 out of 18). Seventeen men reported presenting a treatment supporter when they started their ART, 12 of whom were female. As a result, most of the treatment supporters were women. The ten treatment supporters who were reported as having a continued supportive role over the years, were all women.

### Disclosure and adherence

The reason that many women gave for not having chosen the husband as their supporter, was because of fears of disclosure. Women rarely disclosed their HIV status to partners. Some had tried to disclose by suggesting their partner goes for an HIV-test so that they could then share the test results, but the man had not agreed. Some women who had disclosed to their husbands, reported that the partner was not supportive. For example, there was a 37-year-old woman on the second line ART regimen whose husband left her, and moved to stay with his mother, even though he was the one who had encouraged her to start on ART. Even the women who learned their HIV-positive status after being in long-term relationships feared to disclose. This lack of disclosure affected adherence as there were reports of missing doses for fear of being seen by the partner:

When I am meeting my partner, I do not want him to see me taking the drugs. So when he calls and asks me to meet him, I first give myself time for taking drugs before I start moving, or I take them on my way. It is the morning dose that becomes a problem for I fear him seeing me swallowing drugs, that is when I go beyond the time [for taking the pills]. (female aged 37 years, first line ART, VL <1000 copies/ml, on ART for 4 years)

She added that when she fails to take her pills in the morning she takes both doses (all the five pills) in the evening with ‘lots of water’.

Individuals without treatment supporters appeared to have greater challenges with ART adherence than those who had continued support. Many of those without a supporter had failed on the first line ART regimen or had viral loads greater than 1000 copies/ml: out of the 30 participants with a viral load equal to or greater than 1000 copies/ml, only eight reported continued support from their treatment supporter. For the rest, 10 had never had a supporter while three had never lived with or near the person they had nominated as a supporter and reported getting very little or no help. Four had got support for a few months before the participant or supporter moved away from each other. One woman, for example, had taken her 20 year old nephew with her when she initiated ART to be her treatment supporter, but she felt he had let her down:

The time he would have been useful was when I was weak and could not pick my drugs from the clinic but still, he never helped me pick the drugs from the clinic. Well, I presented him because they told me they were not starting me on ART unless I presented a treatment supporter, for I never lived with him (female aged 43 years, first line ART, VL ≥ 1000 copies/ml, been on ART for 8 years)

A 46 year old man commented about his wife:

She would not mind about me staying alive and would just say let him die. If you have not taken the drugs, it is up to you. (male aged 46 years, second line ART, viral load ≥1000 copies/ml, been on ART for 9 years).

However, he went on to say that while she said this, she also questioned him ‘*Why did you go to take alcohol?*’ suggesting that her lack of support may have stemmed from her frustration at his continued alcohol consumption.

Some, despite the efforts of their treatment supporter, were unable to sustain their health on treatment. Phillip, introduced above, was on re-treatment for Tuberculosis at the time of the interview. During the time of this study he was hospitalised for two months for the initial intensive phase of the TB re-treatment. He had not carried enough ART drugs with him to hospital and he said that what he had with him got finished before he was discharged. It was only when he mentioned his lack of drugs to visitors that ART drugs were taken to him in hospital from the clinic. However, he said that combining ART and TB medication resulted in him having to take six pills a day, which he said he could not manage. So, he stopped taking ART, saying he would complete his TB treatment first and then restart ART. One week after his second interview, we learnt that he had died.

In summary, 23 out the 50 participants talked about the importance of treatment supporters in long-term ART. This number included a few who did not have a current supporter, but mentioned the valuable role a treatment supporter could play during life-long ART. Seventeen participants did not consider a treatment supporter to be necessary in sustaining adherence for long-term ART use. They considered that they were now in the habit of daily pill taking, or recognised the value of the pills for their health so would not default, while others had not had a regular treatment supporter earlier in their time on ART so did not see the need of one once they were well established on ART. Finally, 10 of the participants had no strong preference about whether long-term adherence support was of use or not.

## Discussion

We have shown that the role of treatment supporters often continues to be crucial for people living with HIV after many years on ART. Several participants described the way in which a treatment supporter continued to help them manage their HIV-treatment. We also found that many people without supporters failed on first line ART or had a viral load of or over 1000 copies/ml. A study conducted in South Africa, also showed that people living with HIV, taking ART who had support taking their treatment had suppressed viral loads for longer than those without support (Igumbor et al., 2011).

Mayanja et al. (2013) had previously found for the RCC part of our own study cohort that the lack of a treatment supporter was a hindrance to ART adherence. Research, at a time when ART was still relatively new, had shown that treatment supporters were useful for the first few months on ART but were not essential after six months on treatment (Davies et al., 2006; Foster et al., 2010). It is possible that the importance of long-term support is now being highlighted because people in many countries have been on ART for 10 years or more.

Treatment supporters in our study not only reminded people on ART to take their medication but also helped them with psychological coping. This echoes the findings of other studies where poor mental health, including depression, in people living with HIV and taking ART led to poor or noncompliance with treatment (Akena, Musisi, & Kinyanda, 2010; Kidia et al., 2015; Mutabazi-Mwesigire, Katamba, Martin, Seeley, & Wu, 2015; Nachega et al., 2006). People may also become tired of taking drugs, or busy with other things, and neglect their treatment (Mbonye, Seeley, Ssembajja, Birungi, & Jaffar, 2013); treatment supporters can help in these circumstances.

Women were more likely than men to be treatment supporters. They also appeared to stay in that role longer than men. The greater likelihood of women being treatment supporters than men is in keeping with the role women have played in caring for people living with HIV, throughout the epidemic (Schatz & Ogunmefun, 2007; Schatz & Seeley, 2015; Seeley et al., 1993; Taylor, Seeley, & Kajura, 1996), and in linking relatives, often men, to care (Ahmed et al., 2018; Kawuma, Seeley, Mupambireyi, Cowan, & Bernays, 2018). With the roll out of universal testing and treatment, to provide access to treatment for all people living with HIV, women as treatment supporters may play a crucial role in the provision of HIV-care as health care providers and counsellors manage increasing workloads (Church et al., 2017; Landes et al., 2017). Task shifting to such lay care providers may be essential to support many people living with HIV to link to care (Kennedy, Yeh, Johnson, & Baggaley, 2017; Ruzagira, Baisley, Kamali, & Grosskurth, 2018; Ruzagira, Grosskurth, Kamali, & Baisley, 2017) and stay in care (Hall et al., 2017). These unpaid treatment supporters, however, may need training and support for themselves to enable them to sustain their role in long-term ART care, particularly if that role expands in response to over-stretched health systems.

The preference of women on ART for female treatment supporters rather than their male spouse, may reflect tensions in gender-relations, where women fear disclosing their HIV-status to their partner for fear of the repercussions on the relationship, as a number of studies have documented (see, for example, Karamagi, Tumwine, Tylleskar, & Heggenhougen, 2006; Matovu et al., 2014; Medley, Kennedy, Lunyolo, & Sweat, 2009; Rujumba et al., 2012). However, disclosure to a sexual partner may not have been essential to adherence, if a woman had others she could disclose to and from who she had support. Indeed a number of studies have documented an association between disclosure and improved social support (Gusdal et al., 2011; Kelly et al., 2014; Mbonye et al., 2013; Nachega et al., 2006; Ware et al., 2009).

An important observation on the qualities considered important when choosing a treatment supporter was the issue of proximity to the person living with HIV, taking ART. This appears to be an important determinant of an effective supportive role in the first months of ART before participants’ health improved or before they got used to the daily drug routine. For long-term ART adherence however, the mobility of the person on ART and/or their supporter, placed new demands on the relationship, with treatment supporters being more likely to stay in that role, if they continued to live with, or close by.

## Conclusion

Treatment supporters remain an important psychosocial factor in HIV care in long-term ART treatment. The continued need for a supporter, particularly for people struggling with long-term adherence should be encouraged throughout the treatment, even if that support is intermittent or provided by telephone, rather than face to face. However, that support requires an on-going commitment from those who provide it. The continued role of treatment supporters, many of whom are women, requires recognition of and support for the essential role they play in sustaining relatives and friends on ART.

## Data Availability

The data are archived at the MRC/UVRI and LSHTM Uganda Research Unit, in Entebbe, Uganda. Data access is available in accordance with the MRC/UVRI data access policy. Requests for data can be made to info@mrcuganda.org for the attention of the corresponding author of this paper.
